# Myelin content in older individuals with cerebral small vessel disease: the role of physical activity and lipids

**DOI:** 10.1002/alz70860_104918

**Published:** 2025-12-23

**Authors:** Narlon C. Boa Sorte Silva, Guilherme Moraes Balbim, Ryan G Stein, Yi Gu, Roger C. Tam, Elizabeth Dao, Walid A. Alkeridy, Arthur F. Kramer, Teresa Liu‐Ambrose

**Affiliations:** ^1^ Concordia University, Montréal, QC, Canada; ^2^ University of British Columbia, Vancouver, BC, Canada; ^3^ Centre for Aging SMART, Vancouver Coastal Health Research Institute, Vancouver, BC, Canada; ^4^ Djavad Mowafaghian Centre for Brain Health, Vancouver, BC, Canada; ^5^ King Saud University, Riaydh, Saudi Arabia; ^6^ Beckman Institute, University of Illinois, Urbana, IL, USA; ^7^ Center for Cognitive & Brain Health, Northeastern University, Boston, MA, USA

## Abstract

**Background:**

Physical activity is associated with higher myelin content in older individuals with cerebral small vessel disease (CSVD), a condition marked by demyelination. However, potential mechanisms underlying these associations remain understudied. High‐density lipoprotein (HDL) is involved in remyelination processes within the brain and is modulated by physical activity. Low‐density lipoprotein (LDL) and triglycerides are involved in the pathological mechanisms underlying cerebrovascular diseases. Lipid levels may thus play a significant role in the relationship between physical activity and myelin health.

**Methods:**

We assessed cross‐sectionally whether serum HDL, LDL, and triglycerides moderated the association between physical activity and myelin in older individuals with CSVD and mild cognitive impairment. We performed multiple regression models adjusting for age, sex, education, body mass index, total cholesterol (lipid models only), estimated intracranial volume and white matter lesions (myelin models only). Moderation was tested via an interaction term between physical activity and lipid variables.

**Results:**

We included 81 highly educated, community‐dwelling older individuals (mean age 74.57), 64% of whom were female. Physical activity was positively associated with HDL (standardized B [95% CI] = 0.253 [0.037 to 0.470], *p* = 0.022) as well as with myelin content in the anterior corona radiata (0.225 [0.004 to 0.445], *p* = 0.046), genu of the corpus callosum (0.261 [0.040 to 0.482], *p* = 0.022), and sagittal stratum (0.331 [0.114 to 0.549], *p* = 0.003). HDL was positively associated with myelin content in the body of the corpus callosum (0.344 [0.096 to 0.592], *p* = 0.007, q = 0.037), posterior corona radiata (0.317 [0.097 to 0.537], *p* = 0.005, q = 0.037), superior corona radiata (0.324 [0.095 to 0.552], *p* = 0.006, q = 0.037), and the superior longitudinal fasciculus (0.284 [0.072 to 0.495], *p* = 0.009, q = 0.037). HDL levels significantly moderated the relationship between physical activity and myelin in the sagittal stratum, wherein higher physical activity levels were linked to greater myelin levels for those with average or high HDL (0.289 [0.087 to 0.491], *p* = 0.006).

**Conclusions:**

Physical activity may promote myelin health partly through HDL but data from longitudinal studies are needed to confirm our findings.

